# Variable density and anisotropic field-of-view for 3D Stack-of-Stars radial imaging

**DOI:** 10.1007/s10334-025-01283-x

**Published:** 2025-10-15

**Authors:** Joao Tourais, Guruprasad Krishnamoorthy, Jouke Smink, Marcel Breeuwer, Marc Kouwenhoven

**Affiliations:** 1 Department of Biomedical Engineering, Eindhoven University of Technology, Eindhoven, The Netherlands; 2 Department of MR R&D Clinical Science, Philips, Best, The Netherlands; 3 Department of Imaging Physics, Delft University of Technology, Delft, The Netherlands

**Keywords:** Anisotropic FOV, Elliptical, Variable density, Radial imaging, Stack-Of-Stars

## Abstract

**Objective:**

To develop a non-iterative method for applying elliptical field-of-view (FOV) to radial imaging and evaluate it for Stack-Of-Stars (SOS) with variable radial density in the ${k}_{z}$ direction.

**Materials and methods:**

New analytic expressions were derived to compute the radial profile angles for an elliptical FOV with and without golden angle sampling. With a major-to-minor-axis FOV ratio of 1:0.5, anisotropic FOV and variable density SOS were evaluated, using point spread function analysis, phantom imaging, and in vivo pelvic imaging.

**Results:**

Compared with conventional SOS, elliptical density in ${k}_{z}$ reduced scan time by 20%, while maintaining similar levels of radial aliasing artifacts. Anisotropic FOV reduced scan time by 31%, resulting in similar levels of radial aliasing artifacts at low undersampling for objects with matching in-plane anisotropy. Combining both techniques resulted in a 45% scan time reduction. Alternatively, when compared to conventional SOS using identical scan time, variable density and anisotropic FOV both displayed a lower level of radial aliasing artifacts, although for anisotropic FOV this effect was less pronounced at higher undersampling.

**Discussion:**

Variable density and anisotropic FOV can reduce scan time and/or reduce aliasing artifacts for SOS. The new analytical expressions for elliptical FOV will facilitate future studies on anisotropic FOV radial imaging.

## Introduction

Stack-Of-Stars (SOS) is a 3D acquisition using radial sampling in the in-plane direction (${k}_{x}$-${k}_{y}$) and Cartesian phase encoding in the through-plane direction (${k}_{z}$). Radial imaging oversamples the k-space center, providing robustness against respiratory motion for abdominal, cardiac, and pelvic MRI, as demonstrated for 2D [1] and SOS [2–13]. SOS is often combined with golden angle sampling [14] or pseudo golden angle sampling [9–11], which further reduces motion-induced artifacts since consecutively acquired profiles are spaced by a large angle, resulting in an even distribution of motion effects over k-space [8–15]. To reduce scan time for SOS, conventional parallel imaging techniques [16, 17] and partial Fourier imaging [18] can be applied in the Cartesian slice direction. To further decrease scan time, radial undersampling may be applied, which effectively reduces the in-plane unaliased FOV (uFOV) size [19–21]. When using conventional radial reconstruction with phased array coils, a moderate undersampling generally does not lead to pronounced aliasing artifacts [21], while some applications like contrast-enhanced angiography allow high (up to eightfold) radial undersampling for SOS [4, 19].

With conventional SOS, the angular density of radial profiles is constant along the ${k}_{z}$ axis. Peters et al. [22] proposed variable density sampling for SOS (VSOS), wherein the angular density decreases as a function of $\left|{k}_{z}\right|$. With identical scan times, the radial sampling density in the center of ${k}_{z}$ is higher for VSOS than for conventional SOS, reducing the level of radial aliasing artifacts for VSOS. This was observed for VSOS using an (offset) cosine function [22] and a step function [23]. With an identical radial sampling density in the center of ${k}_{z}$, VSOS results in shorter scan times than conventional SOS [24–26]. VSOS was also combined with golden angle sampling and compressed sensing reconstruction [27–29]. Despite these advantages, variable density is not yet commonly applied for SOS.

Pelvic and abdominal SOS imaging are commonly acquired in axial orientation [2, 3, 8–13]. However, an isotropic (circular) in-plane FOV may result in suboptimal sampling efficiency because in this axial plane, the pelvis/abdomen (including arms) generally has anisotropic dimensions. Scheffler et al. introduced anisotropic FOV for 2D radial imaging [30, 31]. Later, Larson et al. proposed an iterative method to obtain an anisotropic FOV with desired shapes for 2D and 3DPR (projection reconstruction) radial imaging [32]. This method was applied for 2D golden angle sampling (using additional interpolation) [33], for spiral phyllotaxis 3DPR [34], and for UTE 3DPR [35, 36]. By reducing the minor axis FOV, an anisotropic FOV can substantially reduce scan time. With low undersampling, this will not increase the level of radial aliasing for objects with matching anisotropic in-plane dimensions. This was demonstrated for 2D imaging, with a 50% scan time reduction for an elliptical FOV with an anisotropy ratio of 1:0.3 [32]. Alternatively, an anisotropic FOV can reduce the level of aliasing artifacts when compared with an isotropic FOV using identical scan time, as shown for 2D [32, 33] and 3DPR [32, 34]. This effect is more pronounced with low undersampling [32], but it has also been reported with high undersampling [32–34]. Despite these advantages, anisotropic FOV is not commonly applied for radial imaging, including SOS. This may be due to the relative complexity of the required iterative method, compounded by the additional interpolation needed for golden angle sampling. To our knowledge, only three studies have evaluated the benefits of anisotropic FOV for radial imaging [32–34].

While previous studies on radial anisotropic FOV have used cylindrical quadrature coils [32] and (phased array) surface coils [33, 34], the impact of receiver coil (element) size and uniformity on the effectiveness of anisotropic FOV for radial imaging remains unstudied. Similarly, while previous studies on SOS (with a circular FOV) have used phased array surface coils [2–4, 6–11, 19, 21], the effect of receiver coil (element) size on radial aliasing artifacts remains unstudied. We hypothesize that the level of radial aliasing in general, and the effectiveness of anisotropic FOV in particular may depend on the receiver coil.

In this study, we describe a new, non-iterative method to compute the radial profile angles for an elliptical FOV with and without golden angle sampling. This method is applied for Anisotropic FOV SOS (ASOS) and for the combination of Variable density and Anisotropic FOV SOS (VASOS). The effectiveness of VSOS, ASOS and VASOS is assessed through point spread function analysis, phantom imaging, and in vivo pelvic imaging. A phased array coil is used and compared with the quadrature body coil.

## Materials and methods

### Theory

#### Conventional radial imaging and unaliased field-of-view

In radial imaging, the unaliased FOV (uFOV) is determined by the Nyquist criterion [37]. Objects larger than the uFOV will cause aliasing manifested as streaking artifacts in the reconstructed image [21, 38, 39]. The in-plane uFOV for conventional SOS (${uFOV}_{c}$) is defined like the uFOV for 2D radial imaging [19, 30]:

1 $$uFOV_{c} = \frac{{FOV_{r} }}{{N_{r} }} \cdot \frac{2}{\pi } \cdot N_{\theta c}$$

where ${FOV}_{r}$ is the user-defined readout (and reconstruction) FOV size, ${N}_{r}$ the number of corresponding readout samples (acquisition matrix size) on each radial profile, ${FOV}_{r}/{N}_{r}$ the acquired spatial resolution, and ${N}_{\theta c}$ the number of acquired radial profiles (for each ${k}_{z}$ partition). The uFOV has a circular shape with a diameter ${uFOV}_{c}$, and typically ${{uFOV}_{c}\le FOV}_{r}$. For convenience, Eq. 1 can be rewritten as:

2 $$\rho = \frac{{uFOV_{c} }}{{FOV_{r} }} = \frac{2}{\pi } \cdot \frac{{N_{\theta c} }}{{N_{r} }}$$

where $\rho$ is the radial sampling factor ($\rho =1$ for full sampling, $\rho <1$ for undersampling), and $\frac{1}{\rho }$ is the undersampling factor. The total number of acquired profiles is ${N}_{tc}={N}_{\theta c}\cdot {N}_{z}$, where ${N}_{z}$ is the number of acquired ${k}_{z}$ partitions.

#### Variable density sampling for Stack-Of-Stars (VSOS)

**Fig. 1:**
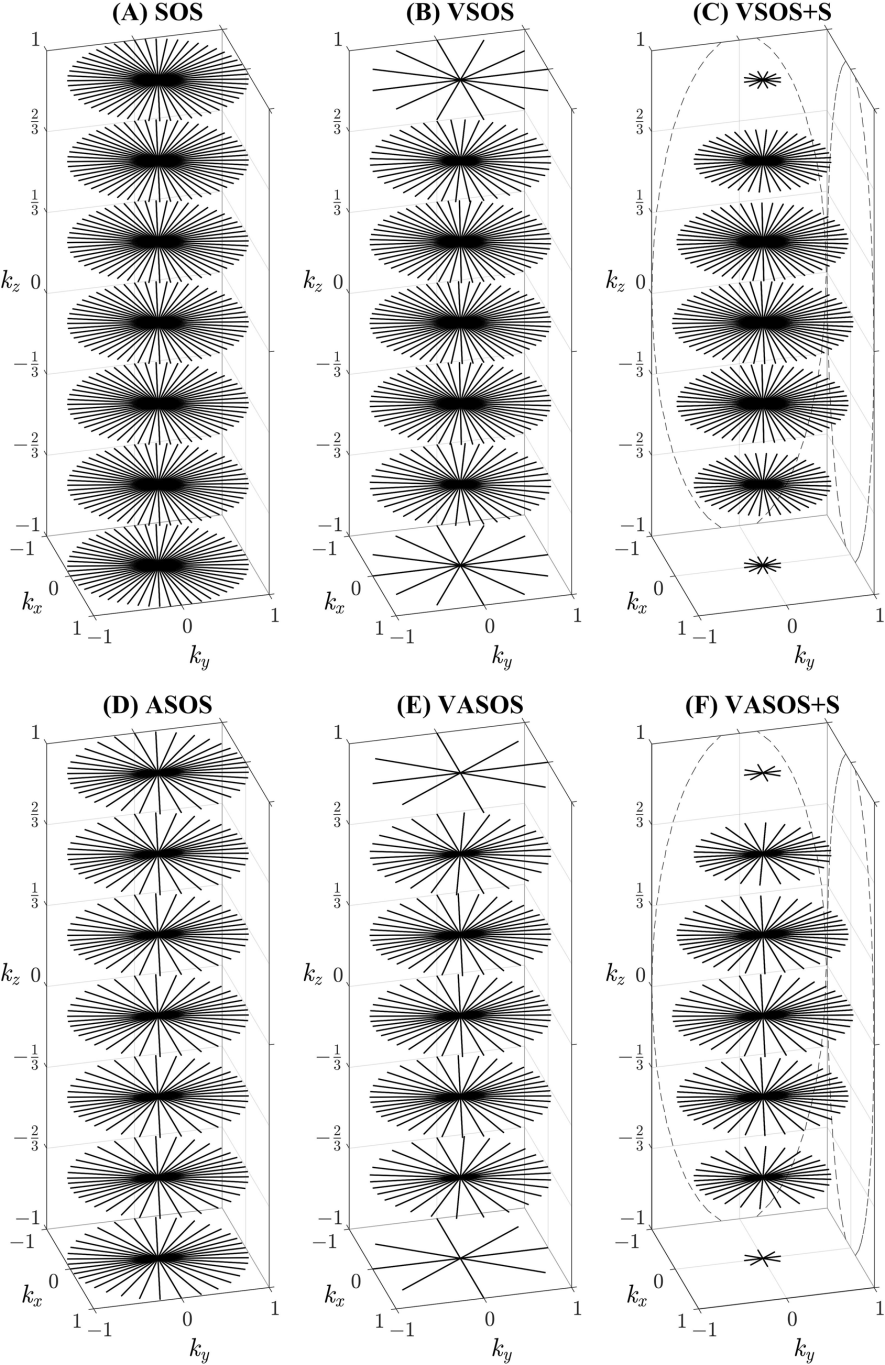
Distribution of radial profiles for six different 3D Stack-Of-Stars (SOS) sampling schemes. **A** Conventional SOS, **B** Variable density SOS (VSOS) without k-space shutter, **C** VSOS with k-space shutter (VSOS + S), **D** Anisotropic FOV SOS (ASOS), **E** Variable density and Anisotropic FOV SOS (VASOS) without k-space shutter and **F** VASOS with k-space shutter (VASOS + S). In **D**–**F**, an elliptical unaliased FOV was used, with an anisotropy $\eta =0.5$ (minor-to-major-axis ratio), and the minor axis in the $y$ direction. An elliptical function (Eqs. 22 and 23 with ${N}_{z}=42$ and ${f}_{p}=1$) was used for the variable density (**B**, **C**, **E**, **F**) and also for the k-space shutter (**C**, **F**). For improved visualization, the irregular spacing of golden angle sampling was not included, only a small number of ${k}_{z}$ partitions and in-plane radial profiles are shown, and the length of the ${k}_{z}$ axis is enlarged with respect to ${k}_{x}$ and ${k}_{y}$

Conventional SOS uses a constant density of radial profiles, where ${N}_{\theta c}$ does not depend on ${k}_{z}$ (Fig. 1a). When a variable density ${D}_{v}\left({k}_{z}\right)$ is applied for SOS (VSOS), the number of radial profiles ${N}_{\theta v}$ will vary over the ${k}_{z}$ partitions (Fig. 1b), and ${N}_{\theta v}\left({k}_{z}\right)$ can be written as:

3 $$N_{\theta v} (k_{z} ) = N_{\theta c} \cdot D_{v} (k_{z} )$$

with ${0<D}_{v}\le 1$, and ${D}_{v}\left(0\right)=1$. For clarity of notation, the parameter ${k}_{z}$ is defined as being normalized $-1\le {k}_{z}\le 1$. The variable density function ${D}_{v}\left({k}_{z}\right)$ should decrease with $\left|{k}_{z}\right|$ but can have several shapes (e.g., elliptical, linear (diamond), step, etc.). Without variable density, ${D}_{v}\left({k}_{z}\right)=1$.

Optionally, a k-space shutter $S\left({k}_{z}\right)$ can be applied, with radius $S$ in the ${k}_{x}$-${k}_{y}$ plane. The number of remaining readout samples (as used for reconstruction) ${N}_{rs}$ will then depend on ${k}_{z}$ and can be expressed as:

4 $$N_{rs} (k_{z} ) = N_{r} \cdot S(k_{z} )$$

with $0<S\le 1$, and $S\left(0\right)=1$. ${FOV}_{r}$ is not affected by the k-space shutter and remains constant over ${k}_{z}$.

When variable density is applied, including the k-space shutter (Fig. 1c), the corresponding uFOV (${uFOV}_{v}$) can be obtained by combining Eqs. 1, 3 and 4:

5 $$uFOV_{v} (k_{z} ) = uFOV_{c} \cdot \frac{{D_{v} (k_{z} )}}{{S(k_{z} )}}$$

where ${uFOV}_{v}(0)={uFOV}_{c}$ is the nominal uFOV for VSOS. The shape of $S\left({k}_{z}\right)$ can be chosen independently from ${D}_{v}\left({k}_{z}\right)$, but in practice $S\left({k}_{z}\right)\ge {D}_{v}\left({k}_{z}\right)$. If no k-space shutter is applied, $S\left({k}_{z}\right)=1$.

**Fig. 2:**
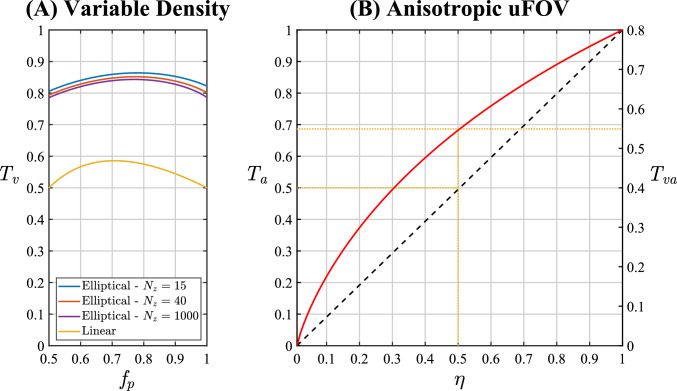
Relative scan time ($T=1/R$, where $R$ is the reduction factor) for variable density, ${T}_{v}\left({f}_{p}\right)$ and for anisotropic unaliased FOV (uFOV), ${T}_{a}\left(\eta \right)$. **A** Relative scan time ${T}_{v}$ (Eq. 6) as a function of the partial Fourier factor (in the slice direction) ${f}_{p}$, for the elliptical variable density function ${D}_{v}$ from Eq. 22, with the number of acquired ${k}_{z}$ partitions ${N}_{z}$ as parameter. For comparison, ${T}_{v}$ is also shown (in yellow) for a linear (diamond) density function (${D}_{v}\left({k}_{z}\right)=1-\left|{k}_{z}\right|$). **B** Relative scan time ${T}_{a}$ (Eq. 16) as a function of the uFOV anisotropy $\eta$ (minor-to-major-axis ratio). The red solid curve is for radial imaging with an elliptical uFOV shape (Eq. 15), whereas the black dashed identity line is for Cartesian imaging with a rectangular (u)FOV (${T}_{a}={FOV}_{y}/{FOV}_{r}=\eta$). The yellow dotted lines show that with $\eta =0.5$, the scan time reduction is 31% for radial (${T}_{a}=0.69$), while for Cartesian it is 50% (${T}_{a}=0.5$). The relative scan time for variable density and anisotropic uFOV combined is ${T}_{va}={T}_{v}\cdot {T}_{a}$ (Eq. 14) and is shown on the right vertical axis of (**B**) for a representative value of ${T}_{v}=0.8$ (${f}_{p}=1$, moderate ${N}_{z}$)

The relative scan time $T$ for VSOS (${T}_{v}$) is defined as the inverse of the reduction factor ($R$) with respect to conventional SOS. ${T}_{v}=\frac{1}{{R}_{v}}=\frac{{N}_{tv}}{{N}_{tc}}=\frac{{\overline{N} }_{\theta v}}{{N}_{\theta c}}={\overline{D} }_{v}$, where ${N}_{tv}$ is the total number of VSOS profiles (${N}_{tv}={\overline{N} }_{\theta v}\cdot {N}_{z}$), ${\overline{N} }_{\theta v}$ the average number of profiles per ${k}_{z}$ partition, and ${\overline{D} }_{v}$ the average variable density (${N}_{\theta v}$ and ${D}_{v}$ averaged over ${k}_{z}$). For a given, even function ${D}_{v}\left({k}_{z}\right)$, ${T}_{v}$ is a function of the partial Fourier factor in the slice direction ${f}_{p}$, and can be expressed as:

6 $$T_{v} (f_{p} ) = \frac{1}{{2f_{p} }}\int\limits_{{ - (2f_{p} - 1)}}^{1} {D_{v} (k_{z} )\,dk_{z} }$$

where $0<{T}_{v}\le 1$ and $0.5<{f}_{p}\le 1$. For example, a linear (diamond) density function ${D}_{v}\left({k}_{z}\right)=1-\left|{k}_{z}\right|$ results in ${T}_{v}\left({f}_{p}\right)=2-{f}_{p}-\frac{1}{2{f}_{p}}$, ${T}_{v}\left(1\right)={T}_{v}\left(\frac{1}{2}\right)=\frac{1}{2}$, and ${T}_{v}\left(\frac{3}{4}\right)=\frac{7}{12}$ (Fig. 2a). Without variable density, ${T}_{v}\left({f}_{p}\right)=1$. The total number of acquired profiles with VSOS is ${N}_{tv}\left({f}_{p}\right)={N}_{z}\cdot {N}_{\theta c}\cdot {T}_{v}\left({f}_{p}\right)$.

#### Anisotropic in-plane field-of-view

**Fig. 3:**
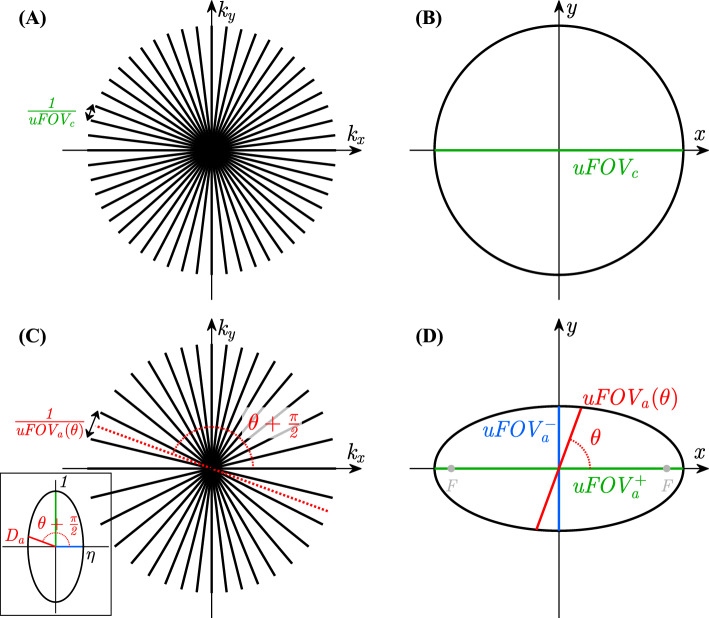
Radial sampling density in k-space and resulting size and shape of the unaliased FOV (uFOV). **A** Radial sampling with isotropic angular density and **B** corresponding circular uFOV with diameter ${uFOV}_{c}$. **C** Radial sampling with anisotropic elliptical angular density ${D}_{a}$ (shown as elliptical radius in the inset) and **D** corresponding elliptical uFOV with diameter ${uFOV}_{a}$ (Eq. 9), minor axis ${uFOV}_{a}^{-}$, and major axis ${uFOV}_{a}^{+}$ (uFOV anisotropy $\eta ={uFOV}_{a}^{-}/{uFOV}_{a}^{+}=0.5$, focal points F with eccentricity ${\eta }^{\prime}=\sqrt{1-{\eta }^{2}}=0.87$). Note that ${uFOV}_{a}\left(\theta \right)\propto {D}_{a}\left(\theta +\frac{\pi }{2}\right)$

For SOS with an isotropic in-plane uFOV, the angular density of radial profiles may depend on ${k}_{z}$ (when variable density is applied), but it is isotropic in the ${k}_{x}$-${k}_{y}$ plane (Figs. 1a–c and 3a). With an Anisotropic in-plane uFOV for SOS (ASOS), the angular density is also anisotropic (${D}_{a}$) and depends on the angle $\theta$ in the ${k}_{x}$-${k}_{y}$ plane (Figs. 1d–f and 3c).

The anisotropic uFOV is denoted as ${uFOV}_{a}$, and its anisotropy $\eta$ is defined as the minor-to-major-axis ratio. The major-to-minor-axis ratio is thus $1\!\!:\!\!\eta$ ($0<\eta \le 1$). The size of the major axis ($\theta =0$, Fig. 3d) is assumed to be unaffected by $\eta$, so $\eta$ is effectively the scale factor of the minor axis $\left(\theta =\frac{\pi }{2}\right)$. Following this definition, and using an isotropic circular uFOV as a reference for ${uFOV}_{a}$ (${uFOV}_{a}={uFOV}_{c}$ for $\eta =1$), its major axis (${uFOV}_{a}^{+}$) and minor axis (${uFOV}_{a}^{-}$) can be defined as:

7 $$uFOV_{a}^{ + } = uFOV_{c}$$

8 $$uFOV_{a}^{ - } \left( \eta \right) = uFOV_{a}^{ + } \cdot \eta$$

The anisotropic density ${D}_{a}$ and the corresponding anisotropic ${uFOV}_{a}$ are a function of $\eta$ and $\theta$. ${uFOV}_{a}$ at angle $\theta$ is proportional to ${D}_{a}$ at perpendicular angle $\theta +\frac{\pi }{2}$ [32] (Fig. 3), and can be expressed as:

9 $$uFOV_{a} \left( {\eta ,\theta } \right) = uFOV_{a}^{ + } \cdot D_{a} \left( {\eta ,\theta + \frac{\pi }{2}} \right)$$

${uFOV}_{a}$ is assumed to be convex, and the density ${D}_{a}\left(\eta ,\theta \right)$ is a $\pi$-periodic function (${D}_{a}\left(\eta ,\theta \right)={D}_{a}\left(\eta ,\theta +\pi \right)$). By definition, ${uFOV}_{a}^{+}\equiv {uFOV}_{a}\left(\eta ,0\right)$, ${uFOV}_{a}^{-}\equiv {uFOV}_{a}\left(\eta ,\frac{\pi }{2}\right)$, ${D}_{a}\left(\eta ,0\right) = \eta$ and ${D}_{a}\left(\eta ,\frac{\pi }{2}\right) = 1$. For a circular uFOV, ${D}_{a}\left(1,\theta \right)=1$, while for a square uFOV, ${1\le D}_{a}\left(1,\theta \right)\le \sqrt{2}$.

The relative scan time for anisotropic FOV is defined as ${T}_{a}=\frac{1}{{R}_{a}}=\frac{{N}_{\theta a}}{{N}_{\theta c}}={\overline{D} }_{a}$, where ${N}_{\theta a}$ is the number of anisotropic profiles (per ${k}_{z}$ partition), and ${\overline{D} }_{a}$ the average anisotropic density (${D}_{a}$ averaged over $\theta$). For a given density function ${D}_{a}\left(\eta ,\theta \right),$ ${T}_{a}$ can be expressed as:

10 $$T_{a} (\eta ) = \frac{1}{\pi }\int\limits_{0}^{\pi } {D_{a}(\eta ,\theta )\,d\theta }$$

where $0<{T}_{a}\le \frac{4}{\pi }$, and ${T}_{a}\left(\eta \right)\ge \frac{2\sqrt{2}}{\pi }\cdot \eta$. For a diamond, circular, or square uFOV ($\eta =1$), ${T}_{a}\left(1\right)=\frac{2\sqrt{2}}{\pi }$, $1$, or $\frac{4}{\pi }$, respectively. For an elliptical uFOV, ${T}_{a}\le 1$, and ${T}_{a}\left(\eta \right)\ge \eta$ (Fig. 2b).

The number of radial profiles (per ${k}_{z}$ partition) for anisotropic uFOV (${N}_{\theta a}$) is a function of $\eta$:

11 $$N_{\theta a} (\eta ) = N_{\theta c} \cdot T_{a} (\eta )$$

#### Variable density and anisotropic field-of-view Stack-Of-Stars (VASOS)

VASOS combines anisotropic in-plane uFOV with variable density (Fig. 1e) and (optional) additional k-space shutter (Fig. 1f). Similar to Eq. 5, the uFOV for VASOS (${uFOV}_{va}$) can be written as:

12 $$uFOV_{va} \left( {k_{z} ,\eta ,\theta } \right) = uFOV_{a} \left( {\eta ,\theta } \right) \cdot \frac{{D_{v} \left( {k_{z} } \right)}}{{S\left( {k_{z} } \right)}}$$

The nominal uFOV for VASOS is ${uFOV}_{va}\left(0,\eta ,\theta \right)={uFOV}_{a}\left(\eta ,\theta \right)$, with major axis ${uFOV}_{va}^{+}\left(0\right)={uFOV}_{a}^{+}$ and minor axis ${uFOV}_{va}^{-}\left(0,\eta \right)={uFOV}_{a}^{-}\left(\eta \right)={uFOV}_{a}^{+}\cdot \eta$.

Similar to Eq. 3, the number of radial profiles (for a given ${k}_{z}$ partition) for VASOS (${N}_{\theta va}$) can be expressed as:

13 $$N_{\theta va} \left( {k_{z} ,\eta } \right) = D_{v} \left( {k_{z} } \right) \cdot N_{\theta a} \left( \eta \right)$$

As shown in the appendix, the relative scan time for VASOS (${T}_{va}$) can be expressed as:

14 $$T_{va} \left( {f_{p} ,\eta } \right) = T_{v} \left( {f_{p} } \right) \cdot T_{a} \left( \eta \right)$$

where $0<{T}_{va}\le \frac{4}{\pi }$. ${T}_{v}$ and ${T}_{a}$ are given by Eqs. 6 and 10, respectively. For an elliptical uFOV, ${T}_{va}\le 1$. For a circular uFOV, and without variable density, ${T}_{va}\left({f}_{p},1\right)=1$.

#### Analytical expressions for elliptical field-of-view profile angles

For an elliptical ${uFOV}_{a}$ with anisotropy $\eta$, in line with Eqs. 7–9, the density ${D}_{a}$ can be written as:

15 $$D_{a} \left( {\eta ,\theta } \right) = \frac{\eta }{{\sqrt {\cos^{2} \left( \theta \right) + \eta^{2} \cdot \sin^{2} \left( \theta \right)} }}$$

where $$\eta \le {D}_{a}\le 1$$, and $${D}_{a}\left(1,\theta \right)=1$$.

With Eqs. 10 and 15, the relative scan time ${T}_{a}\left(\eta \right)$ can be expressed analytically as:

16 $$T_{a} \left( \eta \right){ } = { }\eta \cdot \frac{2}{\pi } \cdot K\left( {\eta^{\prime}} \right)$$

where $K\left(k\right)$ is the complete elliptic integral of the first kind, and ${\eta }^{\prime}=\sqrt{1-{\eta }^{2}}$ is the elliptical eccentricity (usually referred to as “elliptic modulus” with symbol “$k$” in the context of elliptic integrals). ${T}_{a}\left(1\right)=1$ $\left(K\left(0\right)=\frac{\pi }{2}\right)$, ${T}_{a}\, \left(0\right) = 0\,\,\left(\underset{\eta \to 0}{\mathrm{lim}} \,\eta \cdot K\left({\eta }^{\prime}\right)=0\right)$, and for $0<\eta <1$, $\eta <{T}_{a}\left(\eta \right)<1$ (Fig. 2b).

By substituting Eqs. 2 and 16 in Eq. 11, ${N}_{\theta a}\left(\eta \right)$ for ASOS (and 2D imaging) with an elliptical uFOV can be expressed as:

17 $$N_{\theta a} \left( \eta \right) = N_{r} \cdot \rho \cdot \eta \cdot K\left( {\eta^{\prime}} \right)$$

For VASOS with an elliptical uFOV, ${N}_{\theta va}\left({k}_{z},\eta \right)$ is obtained by substituting Eq. 17 in Eq. 13.

**Fig. 4:**
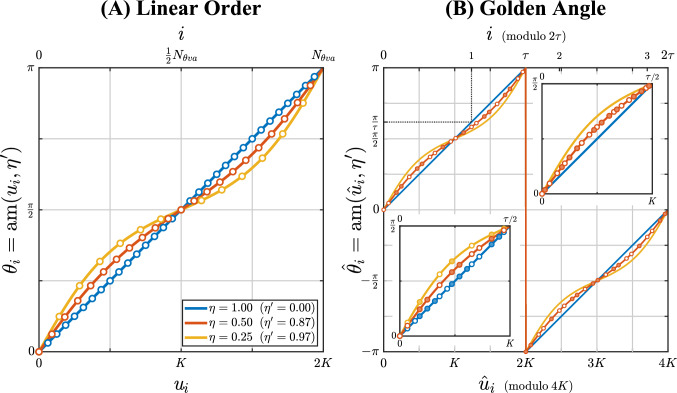
The radial profile angle ${\theta }_{i}={\mathrm{am}}\left({u}_{i},{\eta }^{\prime}\right)$ for an elliptical uFOV as a function of ${u}_{i}$ (in units of $K\left({\eta }^{\prime}\right)$), and as a function of the radial profile index $i$ ($i=\mathrm{0,1},\dots ,{N}_{\theta va}-1)$. For demonstration, only a small number of radial profiles are used, displayed as dots, with ${N}_{\theta va}=32, 22, 14$ for $\eta =1, 0.5, 0.25$, respectively (${T}_{a}\left(\eta \right)=1, 0.69, 0.45$). ${\mathrm{am}}\left({u}_{i},{\eta }^{\prime}\right)$ is the Jacobi elliptical amplitude function, ${\eta }^{\prime}=\sqrt{1-{\eta }^{2}}$ is the elliptical eccentricity, and $\eta$ is the uFOV anisotropy (minor-to-major-axis ratio). $K\left({\eta }^{\prime}\right)$ is the complete elliptical integral of the first kind (for $\eta =1,$ $K\left({\eta }^{\prime}\right)=\frac{\pi }{2})$. **A** ${\theta }_{i}$, ${u}_{i}$ and $i$ for linear profile order (Eqs. 18 and 19) and pseudo golden angle (Eqs. 20 and 34). For each $\eta$, the dots are equidistant in ${u}_{i}$ and $i$. Note that around ${\theta }_{i}=\frac{\pi }{2}$, the angular distance (${\Delta \theta }_{i}$) is similar for all $\eta$, while around ${\theta}_{i}=0$ and $\pi$, ${\Delta \theta }_{i}\propto \frac{1}{\eta }$. **B** ${\hat{theta}}_{i}$, $\hat{u}_{i}$ and $i$ for golden angle sampling (Eqs. 20 and 21), where $\hat{\theta}_{i}$ (${\mathrm{am}}$) is limited (wrapped) from $-\pi$ to $+\pi$. The range for $i$ (modulo $2\tau$) is shown from 0 to $2\tau$, where $i=2\tau$ corresponds with $\hat{u}_{i}=4K$, and $\tau$ is the golden ratio $\tau =\left(1+\sqrt{5}\right)/2=1.618$. The golden angle (${\Delta \hat{\theta }}_{\Delta i=1}={\hat{\theta }}_{1}$ for $\eta =1$) is $\frac{\pi }{\tau }=0.618\cdot \pi$ (black dotted line). The open dots represent the computed radial profile angles. For clarity, dots are only shown for $\eta =0.5 \left({N}_{\theta va}=22\right)$, and solid dots are added as duplicates of the open dots, shifted by $\pi$ for $\hat{\theta}_{i}$ and shifted by $2K$ for $\hat{u}_{i}$ (or by $\tau$ for $i$). Note the irregular dot spacing (in $\hat{u}_{i}$ and $i$) with golden angle. The two insets zoom in on the range $0-K$ ($0-\frac{\tau }{2}$). The bottom left inset displays dots for all three $\eta$, showing the vertical alignment of the dots. The top right inset displays double the number of dots ${(N}_{\theta va}=44$), illustrating the reduced (but still irregular) spacing

As shown in the appendix, for an elliptical FOV, the angle ${\theta }_{i}$ of the $i$^th^ radial profile can be expressed analytically by means of the Jacobi elliptic amplitude function ${\mathrm{am}}\left(u,k\right)$ (Fig. 4a):

18 $$\theta_{i} = {\mathrm{am}}\left( {u_{i} , \eta^{\prime}} \right)$$

where $i=0, 1, 2,\dots , {N}_{\theta va}\left({k}_{z},\eta \right)-1$, ${\theta }_{0}=0$, $0\le {\theta }_{i}<\pi$, and ${u}_{i}$ is defined as:

19 $$u_{i} = \frac{i}{{N_{\theta va} \left( {k_{z} ,\eta } \right)}} \cdot 2K\left( {\eta^{\prime}} \right)$$

The values of ${\theta }_{i}$ and ${u}_{i}$ depend on ${k}_{z}$ and $\eta$ (${\theta }_{i}=\theta \left(i,{k}_{z},\eta \right)$), but for simplicity, this is not included in the notation. For a circular uFOV ($\eta =1$, ${\eta }^{\prime}=0$), Eqs. 18 and 19 reduce to ${\theta }_{i}={u}_{i}=\frac{i}{{N}_{\theta v}\left({k}_{z}\right)}\cdot \pi$, which is a uniform angular distribution for each ${k}_{z}$ partition.

#### Golden angle sampling

For golden angle sampling with VASOS and an elliptical uFOV, the profile angles $\hat{\theta}_{i}$ can also be expressed analytically, similar to Eq. 18:

20 $$\hat{\theta }_{i} = {\mathrm{am}}\left( {\hat{u}_{i} , \eta^{\prime}} \right)$$

where, using the quasi-periodicity of ${\mathrm{am}}$ (${\mathrm{am}}\left(u+2K\left(k\right),k\right)={\mathrm{am}}\left(u,k\right)+\pi$), ${\hat{u}}_{i}$ can be defined as:

21 $$\hat{u}_{i} = \frac{i}{\tau } \cdot 2K\left( {\eta^{\prime}} \right)$$

with $i=0, 1, 2,\dots ,{N}_{\theta va}\left({k}_{z},\eta \right)-1$, $\hat{\theta}_{0}=0$, and the golden ratio $\tau =\left(1+\sqrt{5}\right)/2$. Note that the effective range of ${\hat{\theta}}_{i}$($-\pi <\hat{\theta}_{i}\le \pi$) is twice that of ${\theta }_{i}$, and that the values of $\hat{\theta}_{i}$ and $\hat{u}_{i}$ do not depend on ${k}_{z}$ (Fig. 4b); only the number of profiles per ${k}_{z}$ partition (${N}_{\theta va}$) depends on ${k}_{z}$ (if variable density is applied). For a circular uFOV (${\eta }^{\prime}=0$), Eqs. 20 and 21 reduce to $\hat{\theta }_{i}=\hat{u}_{i}=\frac{i}{\tau }\cdot \pi$, which are the profile angles for conventional golden angle sampling as described by Winkelmann et al. [14].

### Methods

#### Elliptical variable density

For the variable density ${D}_{v}\left({k}_{z}\right)$, an elliptical function was used:

22 $$D_{v} \left( {k_{z} } \right) = \sqrt {1 - \left( {\lambda \cdot k_{z} } \right)^{2} }$$

where $$-1\le {k}_{z}\le 1$$, and

23 $$\lambda = \frac{{N_{z} }}{{N_{z} + f_{p} }} = \frac{{N_{z}^{ + } }}{{N_{z}^{ + } + \frac{1}{2}}}$$

${D}_{v}\left(0\right)=1,$ $0<{D}_{v}\le 1$, $\lambda \le 1$, ${f}_{p}$ is the partial Fourier factor in the slice direction ($0.5<{f}_{p}\le 1$), ${N}_{z}$ the number of acquired ${k}_{z}$ partitions, and ${N}_{z}^{+}={N}_{z}\cdot \frac{1}{2{f}_{p}}$ the number of partitions from ${k}_{z}=0$ to $1$. The factor $\lambda$ is slightly less than 1 and is introduced to ensure that ${D}_{v}\left({k}_{z}\right)>0$ for ${k}_{z}=\pm 1$, which becomes relevant to preserve spatial resolution in the $z$ direction when ${N}_{z}$ is relatively small (e.g., for ${N}_{z}=42$ and ${f}_{p}=1$, $\lambda =0.98$ and ${D}_{v}\left(\pm 1\right)=0.2$, as shown in Figs. 1c and f).

The k-space shutter ($S$) used the same elliptical function as the variable density ($S\left({k}_{z}\right)={D}_{v}\left({k}_{z}\right)$, Eq. 22).

An elliptically shaped ${uFOV}_{a}\left(\eta ,\theta \right)$ was obtained by computing the profile angles ${\theta }_{i}$ for linear order sampling with Eqs. 18 and 19, and $\hat{\theta}_{i}$ for golden angle sampling with Eqs. 20 and 21. The number of profiles ${N}_{\theta va}\left({k}_{z},\eta \right)$ was computed with Eqs. 13 and 17, and subsequently rounded to an integer value. With variable density, ${N}_{\theta va}$ and ${\theta }_{i}$ need to be computed for each ${k}_{z}$ partition, whereas the values of $\hat{\theta}_{i}$ need to be computed only once (for $i=\mathrm{0,1},2,\dots {,N}_{\theta va}\left(0,\eta \right)-1$).

#### Point spread function

The point spread functions (PSF) were calculated in MATLAB (MathWorks, Natick, MA), which contains the $K\left(m\right)$ and ${\mathrm{am}}\left(u|m\right)$ functions ($m={k}^{2}$). For VSOS and VASOS, the PSFs were computed without and with k-space shutter, and they were compared with the PSFs for conventional SOS and ASOS to assess the radial aliasing. The PSFs were computed with linear profile order, ${N}_{r}=300$, ${N}_{z}=84$, ${f}_{p}=1$, $\rho =1$, $\eta =1$ (${N}_{\theta c}={N}_{\theta v}\left(0\right)=471$ for SOS and VSOS), and $\eta =0.5$ (${N}_{\theta a}\left(0.5\right)={N}_{\theta va}\left(0, 0.5\right)=323$ for ASOS and VASOS).

#### MR acquisitions

Phantom and in vivo imaging were performed on a 3T Ingenia scanner (Philips, Best, Netherlands) with R5.7. VASOS acquisition and reconstruction were implemented on the MR scanner software platform. The radial profile angles $\hat{\theta}_{i}$ were computed on the fly during scan definition, using the C++ Boost library v1.59.0 [40] to compute the required $K\left(k\right)$ and ${\mathrm{am}}\left(u,k\right)$. Since ${\mathrm{am}}\left(u,k\right)$ was not directly available in Boost, it was computed with the Jacobi elliptic function ${\mathrm{sn}}$ as:

24 $$\text {am}^{\prime} \left( {u,k} \right) = \arcsin \left( \mathrm{sn}{\left( {u,k} \right)} \right)$$

where $-\pi \le {\mathrm{am}}^{\prime}\le \pi$. In-line radial reconstruction was used, with gridding [41, 42], density compensation [32, 37], phased array coil combination [43, 44], and coil sensitivity-related non-uniformity correction [15, 45]. The angular density compensation function (dcf) ${W}_{\theta }$ is given by:

25 $$W_{\theta } \left( {k_{z} ,\eta ,\theta } \right) = \frac{1}{{D_{va} \left( {k_{z} ,\eta ,\theta } \right)}} = \frac{1}{{D_{v} \left( {k_{z} } \right) \cdot D_{a} \left( {\eta ,\theta } \right)}}$$

where ${D}_{v}\left({k}_{z}\right)$ is given by Eq. 22 and ${D}_{a}\left(\eta ,\theta \right)$ by Eq. 15.

VSOS, ASOS, and VASOS were compared with conventional SOS, and their radial aliasing artifact levels were qualitatively assessed. All acquisitions were repeated using the quadrature body (Q-Body) coil and a phased array surface coil (16-channel anterior array combined with the 12-channel built-in posterior array).

Conventional SOS was acquired in axial orientation using a 3D SOS RF-spoiled gradient echo with golden angle sampling. The scan parameters were: ${FOV}_{r}=550$ mm, volume thickness $=180$ mm, acquisition slice thickness $=3$ mm, acquisition pixel size $=1.5\times 1.5$ mm^2^ (${N}_{r}=367$), reconstruction voxel size $=1.0\times 1.0\times 1.5$ mm^3^, ${f}_{p}=1$ (no partial Fourier), flip angle $=10^\circ$, ${\mathrm{bandwidth}}=1000$ Hz/pixel, and ${\mathrm{TE}}/{\mathrm{TR}}=1.45/3.6$ ms. Adaptive RF shimming was applied [46, 47]. For the phased array coil acquisition, the radial sampling factor was $\rho =0.7$, and SENSE factor $=2$ (in slice direction, with ${N}_{z}=42$), resulting in scan time $=1\mathrm{:}01$ min. For the Q-Body coil acquisition, a larger $\rho$ ($\rho =1.0$) was applied to prevent pronounced radial aliasing, and ${\mathrm{NSA}}=2$ was used to compensate for the inherently much lower SNR, resulting in scan time $=5\mathrm{:}18$ min. ASOS was acquired with $\eta =0.5$ (minor-to-major-axis FOV ratio), but otherwise identical parameters, resulting in a 31% shorter scan time.

The golden angle sampling allowed retrospective undersampling, including variable density (Eq. 22). Hence, VASOS (with and without k-space shutter) and VSOS were reconstructed from ASOS and SOS, respectively, resulting in 20% fewer radial profiles.

The radial sampling factor $\rho$ can be redefined as

26 $$\rho = \rho_{0} \cdot \rho^{\prime}$$

The baseline factor ${\rho }_{0}$ was varied in acquisitions, e.g. comparing different coils. The relative factor $\rho^{\prime}$ is $1.0$ for VSOS, ASOS, VASOS, and conventional SOS (${\mathrm{SOS}}_{0}$). ${\mathrm{SOS}}^{\prime {\mathrm{v}}} ,\,{\mathrm{SOS}}^{\prime {\mathrm{a}}} ,\, \text {and}\,{\mathrm{SOS}}^{\prime {\mathrm{va}}}$ were obtained by retrospectively undersampling ${\mathrm{SOS}}_{0}$ to match the number of radial profiles of VSOS, ASOS, and VASOS, respectively, using $\rho^{\prime} = T_{va}$. Table 1 contains the parameters $\eta ,\,T_{va} ,\,{\mathrm{and}}\,\rho^{\prime}$ for all SOS variants.

**Table 1:** Main parameters for SOS comparisons: uFOV anisotropy $\eta$, relative scan time $T_{va}$, relative radial sampling factor $\rho^{\prime}$, and related factors $\rho^{\prime} \cdot T_{va} ,\frac{1}{{T}_{va}}$, and $\frac{\eta }{{T}_{va}}$.\,\,$\rho^{\prime}<1$ is used for the retrospective undersampling of ${\mathrm{SOS}}_{0}$, to obtain ${\mathrm{SOS}}^{\prime{\mathrm{v}}}$, ${\mathrm{SOS}}^{\prime{\mathrm{a}}}$, and ${\mathrm{SOS}}^{\prime{\mathrm{va}}}$ ($\rho^{\prime}$ is proportional to $uFOV_{c}$ for SOS).

Sampling scheme	$\eta$	${T}_{va}$	${\rho }^{\prime}$	${\rho }^{\prime}\cdot {T}_{va}$	$\frac{1}{{T}_{va}}$	$\frac{\eta }{{T}_{va}}$
${\mathrm{SOS}}_{0}$	1	1	1	1	1	1
VSOS	1	0.8	1	0.8	1.25	1.25
${\mathrm{SOS}}^{\prime{\mathrm{v}}}$	1	1	0.8	0.8	1	1
ASOS	0.5	0.69	1	0.69	1.45	0.72
${\mathrm{SOS}}^{\prime{\mathrm{a}}}$	1	1	0.69	0.69	1	1
VASOS	0.5	0.55	1	0.55	1.82	0.91
${\mathrm{SOS}}^{\prime{\mathrm{va}}}$	1	1	0.55	0.55	1	1

$\rho^{\prime} \cdot T_{va}$ is proportional to the net scan time (Eq. 29) and demonstrates that ${\mathrm{SOS}}^{\prime{\mathrm{v}}}$, ${\mathrm{SOS}}^{\prime{\mathrm{a}}}$, and ${\mathrm{SOS}}^{\prime{\mathrm{va}}}$ have the same scan time as VSOS, ASOS, and VASOS, respectively. $\frac{1}{{T}_{va}}$ is the scale factor for the major axis uFOV of VSOS, ASOS, and VASOS, with respect to the uFOV of the corresponding ${\mathrm{SOS}}^{\prime}$ with the same scan time (${\mathrm{SOS}}^{\prime{\mathrm{v}}}$, ${\mathrm{SOS}}^{\prime{\mathrm{a}}}$, and ${\mathrm{SOS}}^{\prime{\mathrm{va}}}$, respectively). Similarly, $\frac{\eta }{{T}_{va}}$ is the scale factor for the minor axis uFOV

To study the effect of receiver coil (element) size on radial aliasing artifacts, conventional SOS and ASOS were also acquired with a relatively small uFOV ($\rho =0.4$) for both the phased array coil (${\mathrm{NSA}}=1$) and the Q-Body coil (${\mathrm{NSA}}=5$, to compensate for low SNR), and compared with the Q-Body coil acquisition with $\rho =1.0$ (${\mathrm{NSA}}=2$). To study the effect of radial undersampling on ASOS relative to conventional SOS, the phased array coil acquisitions using $\rho =0.4$ and $\rho =0.7$ were compared for ASOS and SOS.

All acquisitions were performed in phantom and in vivo. The phantom setup consisted of a cylindrical image quality phantom and a cylindrical uniform phantom placed side by side, resulting in a total axial cross-section of 190 $\times$ 390 mm^2^. For the in vivo experiments, pelvic images were acquired in two healthy subjects, without applying respiratory compensation. Before scanning, written informed consent was obtained under a protocol approved by the ethics committee.

## Results

Figure 1 shows the distribution of radial profiles for the different SOS sampling schemes (conventional SOS, ASOS, VSOS and VASOS, without and with k-space shutter). Conventional SOS has an angular density of radial profiles that is isotropic in the ${k}_{x}$-${k}_{y}$ plane and constant along the ${k}_{z}$ axis (Fig. 1a). For VSOS and VASOS, the variable angular density decreases towards the periphery of ${k}_{z}$ (Figs. 1b and e). For ASOS and VASOS, the angular density is anisotropic in the ${k}_{x}$-${k}_{y}$ plane (Figs. 1d–f). When the k-space shutter is applied for VSOS and VASOS, the distance between the radial profile tips is constant in ${k}_{z}$ direction, for any given profile angle (Figs. 1c and f).

Figure 2a shows the relative scan time ${T}_{v}$ for elliptical variable density (Eq. 22) as a function of the partial Fourier factor in the slice direction ${f}_{p}$, with the number of acquired ${k}_{z}$ partitions (${N}_{z}$) as parameter. Applying elliptical variable density resulted in a 20% reduction in scan time (${T}_{v}=0.8$) for ${f}_{p}=1$ and a moderate ${N}_{z}$. Figure 2b shows the relative scan time ${T}_{a}$ for an elliptical uFOV (Eq. 16) as a function of the uFOV anisotropy $\eta$. Using an elliptical uFOV with anisotropy $\eta =0.5$ resulted in a 31% reduction in scan time (${T}_{a}=0.69$). Combining the elliptical variable density with the elliptical uFOV resulted in a 45% reduction in scan time for VASOS with $\eta = 0.5\,(T_{va} = T_{v} \cdot T_{a} = 0.8 \cdot 0.69 = 0.55)$.

**Fig. 5:**
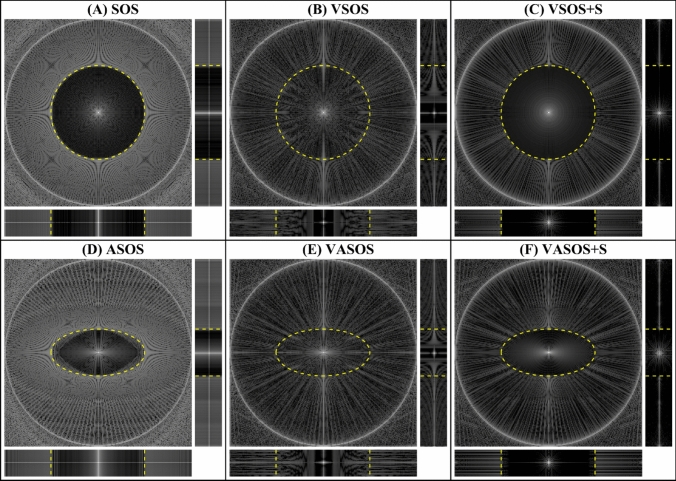
Point Spread Functions (PSFs) for six different Stack-Of-Stars (SOS) schemes. **A** Conventional SOS, **B** Variable density SOS (VSOS) without k-space shutter, **C** VSOS with k-space shutter (VSOS + S), **D** Anisotropic FOV SOS (ASOS), **E** Variable density and Anisotropic FOV SOS (VASOS) without k-space shutter and **F** VASOS with k-space shutter (VASOS + S). For each SOS scheme, the central slices of the transverse (top left), sagittal (top right), and coronal (bottom) orientations are shown. The yellow dashed lines indicate the (nominal) unaliased FOV (uFOV). All PSFs are displayed on a logarithmic gray scale. VSOS (**B** and **C**), ASOS (**D**), and VASOS (**E** and **F**) require 20%, 31%, and 45% fewer profiles compared to conventional SOS (**A**), respectively

Figure 5 shows the PSFs of the different SOS sampling schemes, demonstrating the resulting uFOV and level of radial aliasing artifacts. Conventional SOS (Fig. 5a) and ASOS (Fig. 5d) resulted in a clearly defined aliasing-free cylindroid uFOV. Applying variable density without k-space shutter resulted in an increased level of aliasing inside the nominal uFOV of VSOS (Fig. 5b) and VASOS (Fig. 5e). In the center of the nominal uFOV, a small cylindroid region remained aliasing-free (visible in the sagittal and coronal orientations), with in-plane dimensions determined by the lowest density ${D}_{v}\left({k}_{z}=\pm 1\right)$. Outside the nominal uFOV, the level of aliasing was decreased compared with conventional SOS and ASOS. Applying the k-space shutter for VSOS (Fig. 5c) and VASOS (Fig. 5f) substantially reduced the level of aliasing artifacts inside the (nominal) uFOV, compared with VSOS and VASOS without shutter (Figs. 5b and e).

**Fig. 6:**
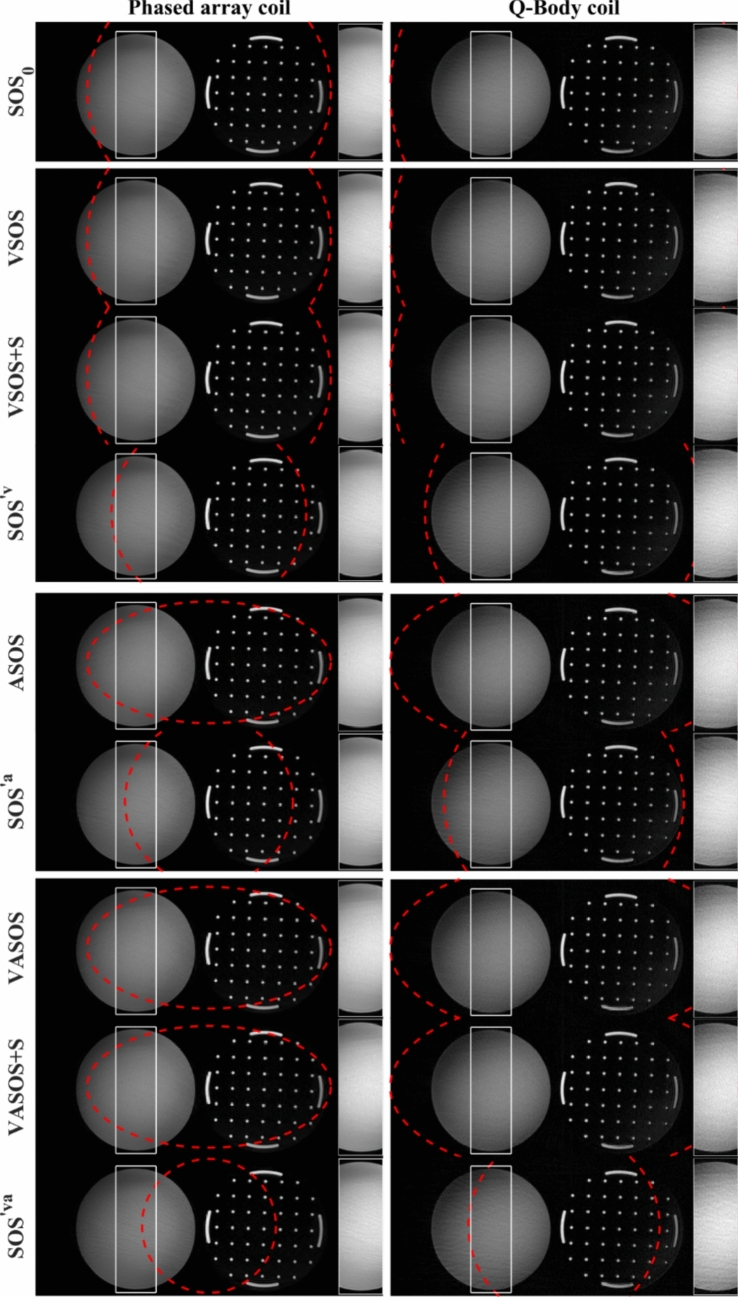
Phantom images comparing conventional SOS, VSOS with (+ S) and without k-space shutter, ASOS, VASOS, and VASOS + S using different unaliased FOV (uFOV) sizes and different coils. The red dashed lines indicate the shape and size of the uFOV. Compared with conventional ${\mathrm{SOS}}_{0}$ (1st row), the scan times for VSOS (2nd and 3rd row), ASOS (5th row), and VASOS (7th and 8th row) were 20%, 31%, and 45% shorter, respectively, while using the same (nominal, major axis) uFOV size. Conventional ${\mathrm{SOS}}^{\prime {\mathrm{v}}}$ (4th row), ${\mathrm{SOS}}^{\prime {\mathrm{a}}}$ (6th row), and ${\mathrm{SOS}}^{\prime {\mathrm{va}}}$ (9th row) had a 20%, 31% and 45% smaller uFOV (${\rho }^{\prime}$) than ${\mathrm{SOS}}_{0}$ and had an identical scan time as VSOS, ASOS, and VASOS, respectively. Phased array coil (left column, ${\rho }_{0}=0.7$) and Q-Body coil with 1.4-fold larger uFOV (right column, ${\rho }_{0}=1.0$). To better illustrate the aliasing artifacts, a region (white rectangle) is enlarged in the inset. Different techniques with the same relative scan time are grouped (separated by a horizontal white line)

**Fig. 7:**
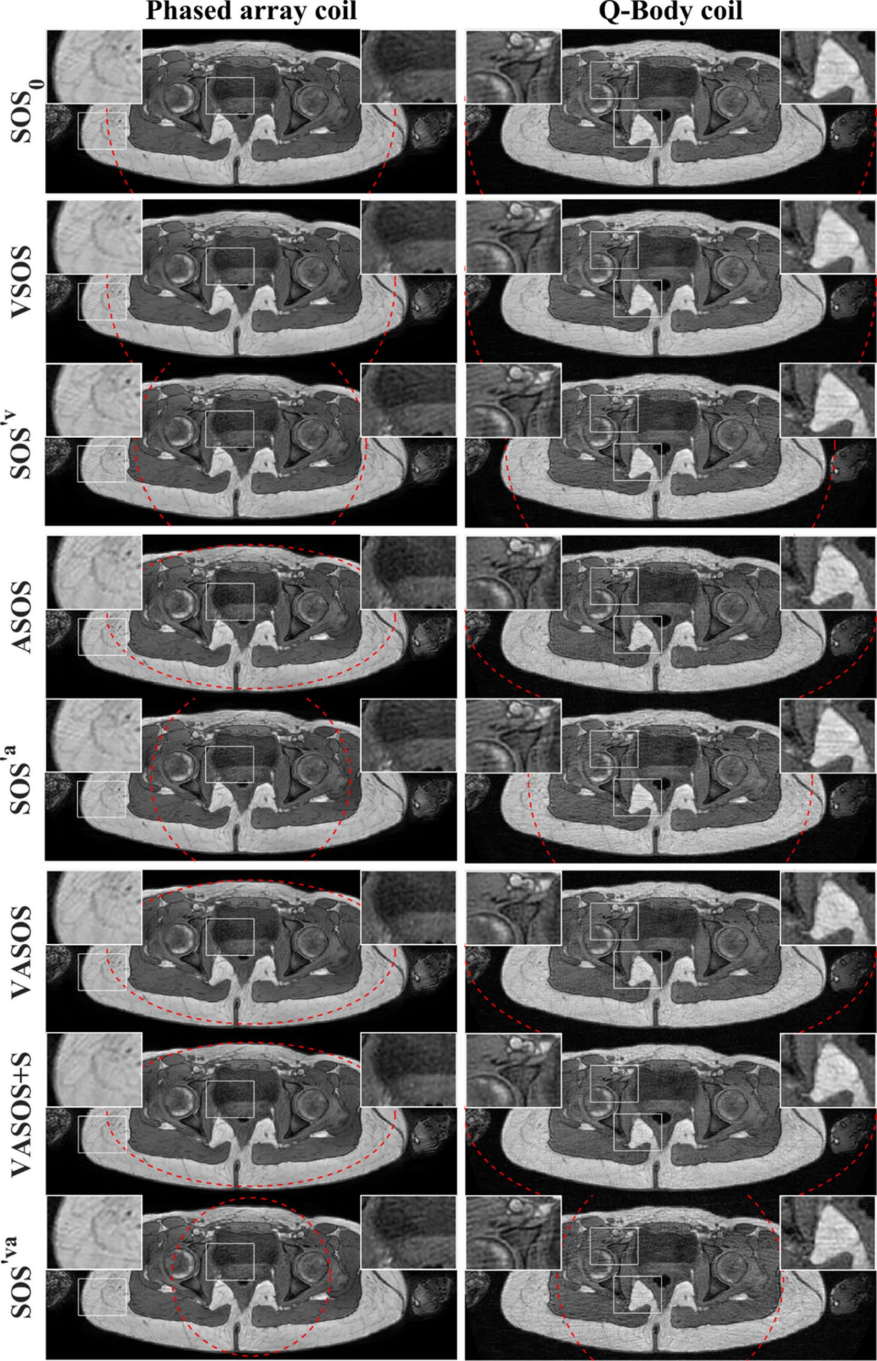
Pelvic images comparing conventional SOS, VSOS, ASOS, and VASOS with (+ S) and without k-space shutter using different unaliased FOV (uFOV) sizes and different coils. The red dashed lines indicate the uFOV. Compared with conventional ${\mathrm{SOS}}_{0}$ (1st row), the scan times for VSOS (2nd row), ASOS (4th row), and VASOS (6th and 7th row) were 20%, 31%, and 45% shorter, respectively, while using the same (nominal, major axis) uFOV size. Conventional ${\mathrm{SOS}}^{\prime {\mathrm{v}}}$ (3rd row), ${\mathrm{SOS}}^{\prime {\mathrm{a}}}$ (5th row), and ${\mathrm{SOS}}^{\prime {\mathrm{va}}}$ (8th row) had a 20%, 31% and 45% smaller uFOV (${\rho }^{\prime}$) than ${\mathrm{SOS}}_{0}$ and had an identical scan time as VSOS, ASOS, and VASOS, respectively. Phased array coil (left column, ${\rho }_{0}=0.7$) and Q-Body coil with 1.4-fold larger uFOV (right column, ${\rho }_{0}=1.0$). To better illustrate the aliasing artifacts, certain regions (white rectangles) are enlarged in the inset. Different techniques with the same relative scan time are grouped (separated by a horizontal white line)

**Fig. 8:**
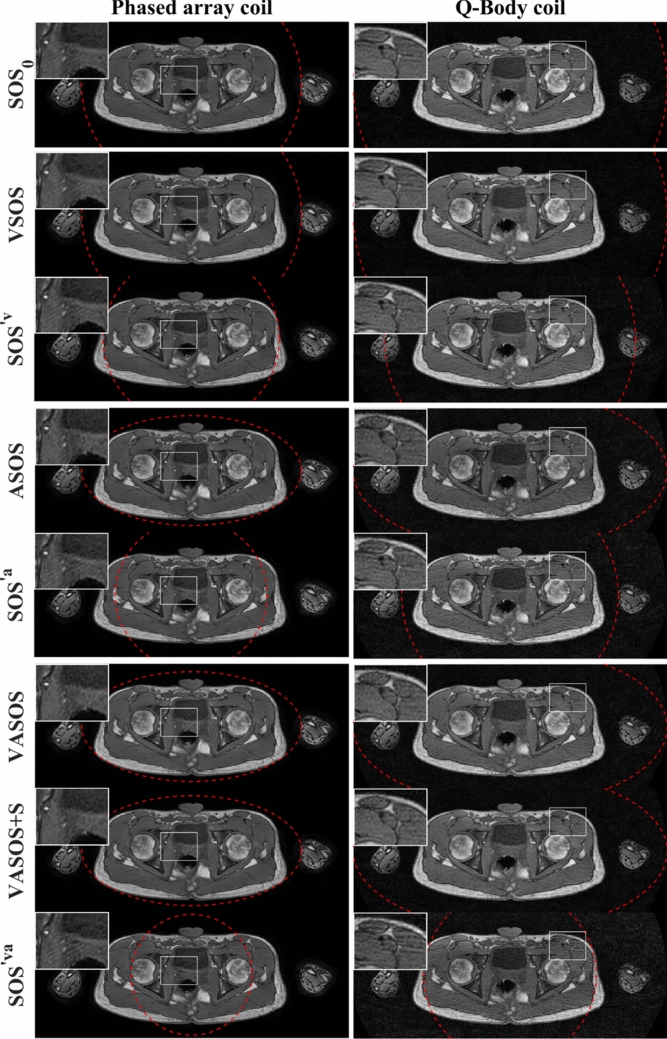
Pelvic images comparing conventional SOS, VSOS, ASOS, and VASOS with (+ S) and without k-space shutter using different unaliased FOV (uFOV) sizes and different coils. The uFOV is indicated by red dashed lines. Compared with conventional ${\mathrm{SOS}}_{0}$ (1st row), the scan times for VSOS (2nd row), ASOS (4th row), and VASOS (6th and 7th row) were 20%, 31%, and 45% shorter, respectively, while using the same (nominal, major axis) uFOV size. Conventional ${\mathrm{SOS}}^{\prime {\mathrm{v}}}$ (3rd row), ${\mathrm{SOS}}^{\prime {\mathrm{a}}}$ (5th row), and ${\mathrm{SOS}}^{\prime {\mathrm{va}}}$ (8th row) had a 20%, 31% and 45% smaller uFOV (${\rho }^{\prime}$) than ${\mathrm{SOS}}_{0}$ and had an identical scan time as VSOS, ASOS, and VASOS, respectively. Phased array coil (left column, ${\rho }_{0}=0.7$) and Q-Body coil with a 1.4-fold larger uFOV (right column, ${\rho }_{0}=1.0$). To better illustrate the aliasing artifacts, a region (white rectangle) is enlarged in the inset. Different techniques with the same relative scan time are grouped (separated by a horizontal white line)

Figures 6, 7, and 8 compare the different SOS sampling schemes, using a phased array coil $(\rho_{0} = 0.7)$ and the Q-Body coil $(\rho_{0} = 1.0)$, for phantom and in vivo imaging. VSOS, ASOS, and VASOS showed similar aliasing artifact levels as conventional SOS (${\mathrm{SOS}}_{0}$) using the same (nominal, major axis) uFOV size, while requiring 20%, 31%, and 45% fewer profiles due to variable density, reduced minor axis uFOV, and both combined, respectively. VSOS, ASOS, and VASOS showed a lower level of radial aliasing artifacts compared with conventional SOS using the same scan time (${\mathrm{SOS}}^{\prime {\mathrm{v}}} ,\,{\mathrm{SOS}}^{\prime {\mathrm{a}}} ,\, \text {and}\,{\mathrm{SOS}}^{\prime {\mathrm{va}}}$, with a 20%, 31% and 45% smaller uFOV than ${\mathrm{SOS}}_{0}$, respectively). VASOS with k-space shutter (VASOS + S) showed similar aliasing artifact levels as VASOS without k-space shutter. All these results were observed for both the phased array coil and the Q-Body coil, although for the phased array coil, the effect of anisotropic FOV was less pronounced. Additionally, for all types of SOS sampling schemes, the phased array coil showed lower radial aliasing levels than the Q-Body coil, despite using a larger uFOV ($\rho$) for the latter.

**Fig. 9:**
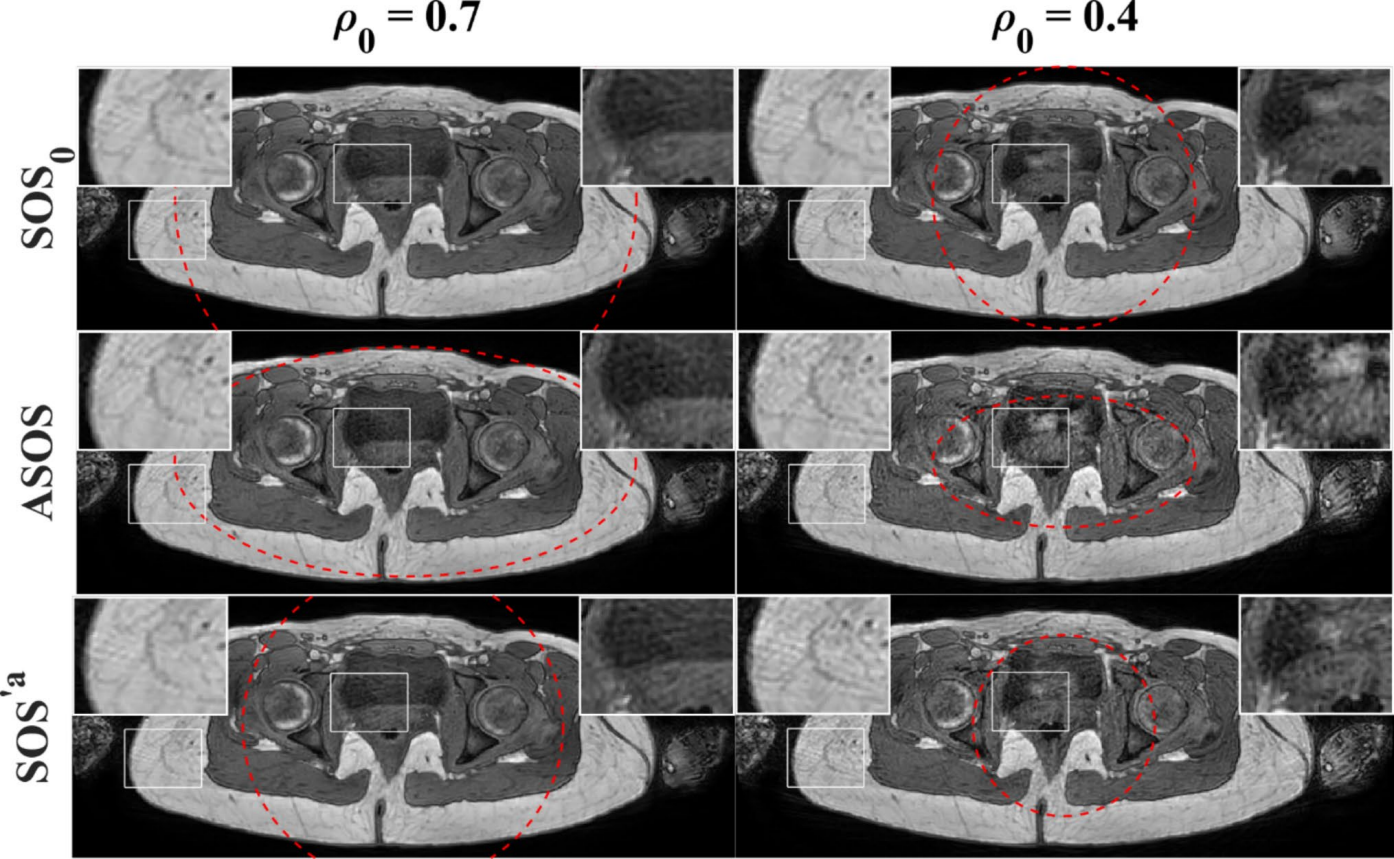
Pelvic images comparing sampling factors ${\rho }_{0}=0.7$ (left column,) and ${\rho }_{0}=0.4$ (right column) for conventional SOS (${\mathrm{SOS}}_{0}$) and ASOS acquisitions, using the phased array coil. ${\mathrm{SOS}}^{\prime {\mathrm{a}}}$ was retrospectively undersampled from ${\mathrm{SOS}}_{0}$. The red dashed lines indicate the size and shape of the uFOV. ASOS (middle row) had a 31% shorter scan time than ${\mathrm{SOS}}_{0}$ (top row), while having the same (major axis) uFOV size. ${\mathrm{SOS}}^{\prime {\mathrm{a}}}$ (bottom row) had the same scan time as ASOS, while having a 31% smaller uFOV (${\rho }^{\prime}=0.69$) than ${\mathrm{SOS}}_{0}$. The images in the left column are identical to the corresponding images in Fig. 7. To better illustrate the aliasing artifacts, certain regions (white rectangles) are enlarged in the inset

Figure 9 shows the effect of radial undersampling on ASOS relative to conventional SOS, for the phased array coil. For $\rho =0.7$, ASOS showed a similar level of artifacts as conventional SOS with the same (major axis) uFOV size (${\mathrm{SOS}}_{0}$), as already shown in Figs. 6–8, while for $\rho =0.4$, ASOS showed a higher level of artifacts than ${\mathrm{SOS}}_{0}$. For both $\rho =0.7$ and $\rho =0.4$, ASOS showed a reduced aliasing artifact level compared with conventional SOS using the same scan time (${\mathrm{SOS}}^{\prime {\mathrm{a}}}$, with a 31% smaller uFOV than ${\mathrm{SOS}}_{0}$). However, for ASOS with $\rho =0.4$, this reduction in aliasing was less pronounced than for ASOS with $\rho =0.7$.

**Fig. 10:**
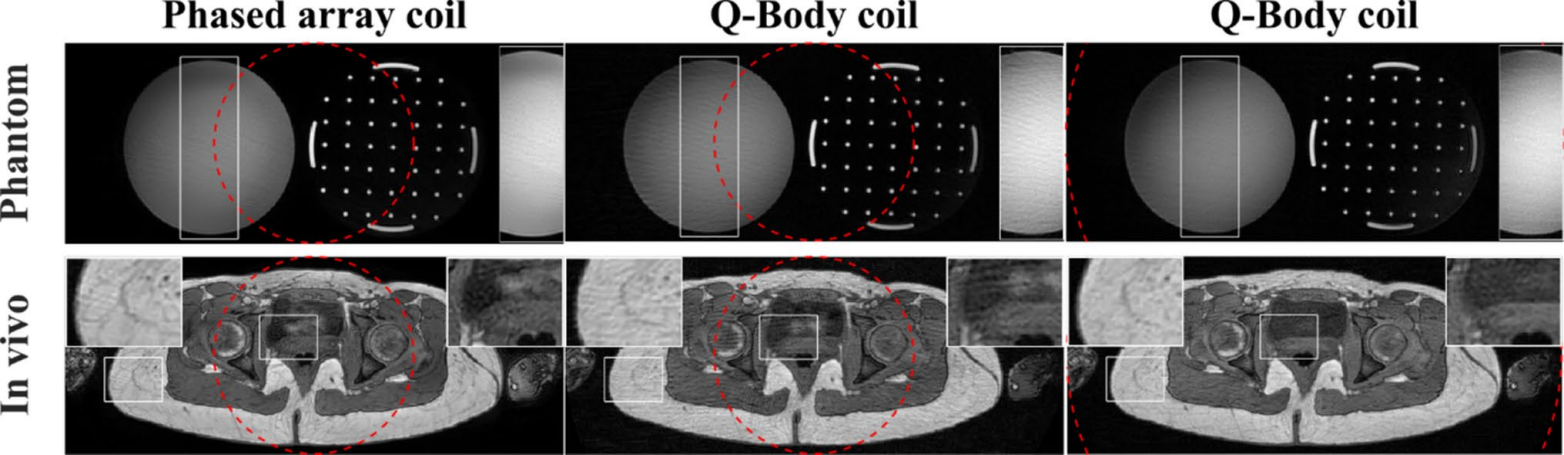
Phantom (top row) and pelvic (bottom row) images obtained using conventional SOS with different coils and unaliased FOV (uFOV) sizes. The red dashed lines indicate the uFOV size. Phased array coil (left column, $\rho =0.4$), Q-Body coil with the same uFOV (middle column, $\rho =0.4$), and Q-Body coil with 2.5-fold larger uFOV (right column, $\rho =1$). To compensate for the lower SNR with the Q-Body coil, ${\mathrm{NSA}}=5$ was used for $\rho =0.4$, and ${\mathrm{NSA}}=2$ for $\rho =1$. To better illustrate the aliasing artifacts, certain regions (white rectangles) are enlarged in the inset

Figure 10 shows the effect of receiver coil on radial aliasing. The phased array coil acquisitions showed a substantially lower level of aliasing than Q-Body coil acquisitions with the same uFOV, while showing a similar level of aliasing as the Q-Body coil acquisitions with a 2.5-fold larger uFOV.

## Discussion

### Analytic expressions for elliptical uFOV profile angles

New, analytic expressions are proposed to compute the radial profile angles ${\theta }_{i}$ and the number of profiles ${N}_{\theta a}$ for an elliptical uFOV shape (Eqs. 17–19). These expressions offer a convenient alternative to the iterative method proposed by Larson et al. [32], although the latter is more general by allowing any convex uFOV shape.

The new analytic expressions also include the profile angles $\hat{\theta}_{i}$ for golden angle sampling with elliptical uFOV (Eqs. 20 and 21), eliminating the additional requirement for interpolation as described by Wu et al. [33]. Pseudo golden angle sampling [9–11] is also supported (Eqs. 20 and 34). Tiny golden angle sampling [48, 49] can be obtained by substituting $\tau$ in Eq. 21 or 34 with $\tau^{\prime} = \tau + M - 1,$ where $M=1, 2, 3,\dots$, with $M=2$ being the complementary small golden angle sampling, and $M\ge 3$ the tiny golden angle sampling.

Besides SOS, the analytic expressions for an elliptical uFOV are also applicable for 2D and 3DPR.

Eqs. 17–21 and 34 use the complete elliptic integral of the first kind $K\left(k\right)$ and the Jacobi elliptic amplitude function ${\mathrm{am}}\left(u,k\right)$, which can be computed with many software libraries and platforms (e.g., C++ Boost, Python SciPy, MATLAB, and Mathematica).

### Variable density

As shown in Fig. 2a, the elliptical variable density function (Eq. 22) allows a maximum scan time reduction of 21.5% $\left( {T_{v} = \frac{\pi }{4}} \right)$, if no partial Fourier is applied (${f}_{p}=1$) and if a large number of ${k}_{z}$ partitions (${N}_{z}$) is acquired. A moderate ${N}_{z}$, as used for phantom and in vivo imaging (with ${f}_{p}=1$), resulted in a slightly smaller scan time reduction of 20% (${T}_{v}=0.8$). Other density functions can be applied (e.g., cosine [22], step [23], linear (diamond) [25]), which may achieve greater scan time reductions at the cost of increased aliasing for peripheral ${k}_{z}$ partitions.

When variable density is applied without k-space shutter ($S\left({k}_{z}\right)=1$), the uFOV $\mathrm{s}$ize depends on ${k}_{z}$ and is proportional to the density function ${D}_{v}\left({k}_{z}\right)$ (Eqs. 5 and 12) which decreases towards the periphery of ${k}_{z}$. This resulted in an increased aliasing artifact level inside the nominal uFOV of the PSF for VSOS compared with SOS (Fig. 5b vs a) and VASOS compared with ASOS (Fig. 5e vs d). As a result of this aliasing induced by variable density, the level of pseudo-noise slightly increased in phantom imaging (Fig. 6, VSOS vs ${\mathrm{SOS}}_{0}$ and VASOS vs ASOS). When additionally, a k-space shutter is applied using the same function as the variable density (${S\left({k}_{z}\right)=D}_{v}\left({k}_{z}\right)$), the uFOV no longer depends on ${k}_{z}$ (Eqs. 5 and 12) and is equal to the nominal uFOV. This eliminated the aliasing induced by variable density in the PSF (Figs. 5c and f) and slightly reduced the pseudo-noise in phantom imaging (Fig. 6, VSOS + S and VASOS + S). Applying an elliptical k-space shutter produces a spheroid boundary in k-space, resulting in a more isotropic spatial resolution [50], similar to the spherical Stack-Of-Spirals trajectory [51, 52].

The PSF showed a reduced aliasing artifact level outside the nominal uFOV for VSOS and VASOS (Figs. 5b,c,e,f) compared with conventional SOS and ASOS (Figs. 5a,d). This reduction is due to the linear profile order as used for the PSF, which results in the nonalignment of profiles throughout ${k}_{z}$ when combined with variable density, similar to the effect observed with rotated SOS [53].

### Anisotropic FOV and scan time

As shown in Fig. 2b, for radial imaging with an anisotropic elliptical uFOV, the relative scan time ${T}_{a}$ is non-linear with the FOV anisotropy $\eta$, and ${T}_{a}>\eta$ (for $\eta <1)$, while for Cartesian imaging, ${T}_{a}=\eta$ ($\eta ={FOV}_{y}/{FOV}_{r}$). Thus, the scan time reduction ($1-{T}_{a}$) for radial imaging is smaller than for Cartesian. For example, with $\eta =0.5$, for radial imaging with an elliptical uFOV, scan time is reduced by 31% (${T}_{a}=0.69$) instead of 50% for Cartesian. By combining the elliptical uFOV with elliptical variable density, a higher scan time reduction of 45% $(T_{va} = T_{v} \cdot T_{a} = 0.55)$ is obtained for VASOS.

### Impact of coil (element) size

To evaluate the impact of coil (element) size on the aliasing level, conventional SOS and ASOS were acquired with a relatively small uFOV ($\rho =0.4$) for both the Q-Body coil and the phased array coil (Fig. 10). The phased array coil showed a substantially lower level of aliasing artifacts than the Q-Body coil. This result contrasts with conventional Cartesian imaging (without parallel imaging), where aliasing artifacts with Q-Body and phased array coils are typically similar [54, 55]. Radial sampling uses a rotating readout gradient with oversampling, resulting in a relatively low level of aliasing artifacts for the images of each single element from the phased array coil, due to their relatively small size and corresponding sensitivity area. After coil combination, this results in a low aliasing artifact level in the final image. This is similar to the behavior of aliasing artifacts in Cartesian Parallel Imaging with Localized Sensitivity (PILS) [55, 56]. However, to obtain an aliasing-free image, PILS requires a uFOV smaller than the width of the coil element sensitivity and the knowledge of the coil element positions during reconstruction, which is not required for radial.

**Fig. 11:**
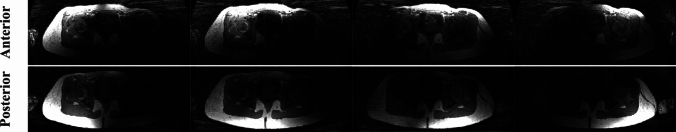
Eight single-element images from the phased array coil. Four anterior coil elements (top row) and four posterior coil elements (bottom row) are displayed. Pelvic imaging was performed in axial orientation using conventional SOS (${\mathrm{SOS}}_{0}$ acquisition from Fig. 7)

For each coil, VSOS, ASOS, and VASOS resulted in a lower radial aliasing artifact level compared with conventional SOS using identical scan time. As expected, the effect of variable density was independent of the used coil. However, the effect of anisotropic FOV was less pronounced with the phased array coil compared with the Q-Body coil, consistent with findings from previous studies using cylindrical quadrature coils [32] and (phased array) surface coils [33, 34]. This difference may be attributed to the non-uniform and relatively small sensitivity area of each phased array coil element (Fig. 11), which effectively reduces the impact of object anisotropy, while the Q-Body coil has a uniform sensitivity throughout the entire anisotropic object. Nevertheless, the phased array coil still showed a positive effect for anisotropic FOV because, despite the non-uniformity, each coil element is still sensitive to a major part of the object (particularly the central elements). Moreover, the contours of constant sensitivity for each coil element are anisotropic in planes perpendicular to the coil [57], with their larger dimensions aligned parallel to the major axis of the scanned object in axial imaging. This is also relevant for 3DPR with selective excitation and anisotropic unaliased FOV in the slice direction. When the excited 3D volume thickness is relatively thin (roughly equal or smaller than the coil element size in that direction), applying an anisotropic, oblate unaliased FOV can be beneficial [32, 34].

### Anisotropic FOV and undersampling

The effectiveness of anisotropic FOV may depend on the level of undersampling. At low undersampling levels, reducing the minor axis unaliased FOV decreases scan time without increasing the level of radial aliasing (for objects with matching anisotropic in-plane dimensions), while at higher undersampling, tailoring the unaliased FOV using the same scan time reduces the level of radial aliasing, as previously reported by Larson et al. [32]. As shown in Fig. 9, the overall reduction in aliasing artifact level of ASOS compared with conventional SOS using identical scan time ${\mathrm{SOS}}^{{^{\prime}}^{a}}$ was fairly pronounced at lower undersampling levels (large ${\rho }_{0}$), while it appeared less pronounced at higher undersampling levels (smaller ${\rho }_{0}$). This is to be expected, because for ASOS with low undersampling, the corresponding ${\mathrm{SOS}}^{{^{\prime}}^{a}}$ oversampled the object in the vertical direction, while for ASOS with higher undersampling, the corresponding ${\mathrm{SOS}}^{{^{\prime}}^{a}}$ undersampled the object in this direction. However, even for the higher undersampling, ASOS still showed some benefits compared to the corresponding ${\mathrm{SOS}}^{{^{\prime}}^{a}}$. This result is in line with earlier studies, which found benefits of anisotropic FOV even in the case of highly undersampled radial acquisitions, for 2D [33] and 3DPR [32, 34].

### Acceleration techniques

Radial parallel imaging [58–60], radial compressed sensing [8, 20, 61–65], and deep learning-based reconstructions [66, 67] might be combined with variable density and anisotropic in-plane FOV SOS. Whereas the potential benefits of anisotropic in-plane FOV for these radial acceleration techniques still need to be investigated, variable density has already shown benefits for radial compressed sensing [29]. Cartesian compressed sensing commonly employs variable density random undersampling, with a lower sampling density at the periphery of k-space than at the center [69]. Variable density SOS results in a decreasing radial sampling density towards the periphery of ${k}_{z}$, and is thus beneficial for compressed sensing. This has already been demonstrated using a density step function for VSOS [29]. Sampling incoherence in the ${k}_{z}$ direction also benefits compressed sensing, and this can be obtained with rotated SOS [53] or with a variable density of ${k}_{z}$ planes [68]. Additionally, combining an elliptical k-space shutter with compressed sensing (using $S\left({k}_{z}\right)>{D}_{v}\left({k}_{z}\right)$) may be beneficial because it could reduce image noise without compromising spatial resolution.

The effects of anisotropic FOV on image quality can be fairly subtle, and aliasing artifacts may be partly visible as pseudo-noise. To investigate the effects with sufficiently high SNR, our study only used through-plane acceleration, with a moderate acceleration factor of 2 for the phased array coil. For the Q-Body coil, even without acceleration, 2–5 signal averages were necessary to be able to properly study the effects. Also, at the time of our study, in-plane radial acceleration reconstruction techniques were not yet available on the scanner.

Furthermore, compressed sensing with radial undersampling has high computational demands for reconstruction [20, 21, 69], while VASOS provides an alternative method for acceleration with a reduction factor of almost 2, without an increase in computational demand.

Although our study did not include radial in-plane acceleration techniques, it provides relevant insights into the effects of radial undersampling and it is the first to evaluate the benefits of anisotropic FOV for SOS. Additionally, our newly developed analytical expressions for elliptical FOV radial profile angles - with and without golden angle - will facilitate future studies in assessing the potential benefits of anisotropic FOV for radial imaging with in-plane acceleration techniques.

### Conclusions

The effectiveness of variable density and anisotropic FOV was evaluated for 3D Stack-Of-Stars radial imaging using an elliptical variable density function and an elliptical in-plane uFOV with a major-to-minor-axis ratio of 1:0.5. The impact of receiver coil on the effectiveness of these techniques was also studied by comparing a phased array coil with the quadrature body coil.

Compared with conventional SOS, variable density reduced scan time by 20% while maintaining comparable levels of radial aliasing artifacts. Anisotropic FOV decreased scan time by 31%, achieving similar radial aliasing artifact levels at low undersampling, for objects with matching in-plane anisotropy. Combining both techniques resulted in a 45% scan time reduction. Alternatively, when comparing with conventional SOS using identical scan time, variable density and anisotropic FOV both reduced the radial aliasing artifact level, albeit less pronounced at higher undersampling for anisotropic FOV.

All these results were observed for both the Q-Body coil and the phased array coil, even though the phased array coil generally resulted in a lower aliasing artifact level than the Q-Body coil. The benefits of variable density were independent of the used coil, while the benefits of anisotropic FOV were less pronounced with the phased array coil.

We have presented new analytic expressions to compute the profile angles for radial imaging with an elliptical uFOV, including golden angle sampling. This will facilitate future studies on radial imaging with anisotropic FOV, including in-plane acceleration techniques.

## Data Availability

The data that support the findings of this study are not openly available due to reasons of sensitivity and are available from the corresponding author upon reasonable request.

## Appendix

### *Profile density *${D}_{va}$*, relative scan time *${T}_{va}$* and number of profiles *${N}_{tva}$* for VASOS*

The variable, anisotropic radial profile density for VASOS (${D}_{va}$) is a function of ${k}_{z}$, $\eta$ and $\theta$, and is the product of ${D}_{v}$ and ${D}_{a}$:

27 $$D_{va} \left( {k_{z} ,\eta ,\theta } \right) = D_{v} \left( {k_{z} } \right) \cdot D_{a} \left( {\eta ,\theta } \right)$$

The relative scan time for VASOS (${T}_{va}$) is defined as ${T}_{va}=\frac{1}{{R}_{va}}=\frac{{N}_{tva}}{{N}_{tc}}={\overline{D} }_{va}$, where ${N}_{tva}$ is the total number of VASOS profiles, and ${\overline{D} }_{va}$ the average density (${D}_{va}$ averaged over both ${k}_{z}$ and $\theta$). Because ${D}_{v}$ and ${D}_{a}$ are independent functions, $\overline{D}_{va} = \overline{D}_{v} \cdot \overline{D}_{a}$, and ${T}_{va}$ can be expressed as:

28 $$T_{va} \left( {f_{p} ,\eta } \right) = T_{v} \left( {f_{p} } \right) \cdot T_{a} \left( \eta \right)$$

where $0<{T}_{va}\le \frac{4}{\pi }$. ${T}_{v}$ and ${T}_{a}$ are given by Eqs. 6 and 10, respectively. For an elliptical uFOV, ${T}_{va}\le 1$. For a circular uFOV, and without variable density, ${T}_{va}\left({f}_{p},1\right)=1$. The total number of acquired profiles with VASOS is ${N}_{tva}\left({f}_{p},\eta \right)={N}_{z}\cdot {N}_{\theta c}\cdot {T}_{va}\left({f}_{p},\eta \right)$. With Eq. 2, this can be written as:

29 $$N_{tva} \left( {f_{p} ,\eta } \right) = \frac{{N_{z} \cdot N_{r} \cdot \pi }}{2} \cdot \rho \cdot T_{va} \left( {f_{p} ,\eta } \right)$$

where the radial sampling factor $\rho ={uFOV}_{c}/{FOV}_{r}={uFOV}_{a}^{+}/{FOV}_{r}$ (using Eq. 7).

### *Profile angles *${\theta }_{i}$* for elliptical FOV*

Wu et al. [33] used the iterative method from Larson et al. [32] to compute the radial profile angles $$\theta$$ for anisotropic uFOV for 2D imaging. They also already noticed that the angle $${\theta }_{i}$$ of the $$i$$^th^ radial profile is determined by the integral from 0 to $${\theta }_{i}$$ of the anisotropic profile density, which is proportional to the number of profiles in that range. For each $${k}_{z}$$ partition in VASOS, with a linear profile order, this means that $$\mathop \int \limits_{0}^{{\theta_{i}}}{D_{va} (k_{z} ,\eta ,\theta )} \,d\theta \, \propto \,i$$$$.$$ With Eq. 27, this can be expressed as:

30 $$\mathop \int \limits_{0}^{{\theta_{i} }} D_{a} \left( {\eta ,\theta } \right)\, d\theta = \frac{i}{{N_{\theta va} \left( {k_{z} ,\eta } \right)}} \cdot \mathop \int \limits_{0}^{\pi } D_{a} \left( {\eta ,\theta } \right)\,d\theta$$

where $i=0, 1, 2,\dots , {N}_{\theta va}\left({k}_{z},\eta \right)-1$, ${\theta }_{0}=0$, and $0\le {\theta }_{i}<\pi$ (by definition, ${\theta }_{{N}_{\theta va}\left({k}_{z},\eta \right)}=\pi$). ${\theta }_{i}$ depends on ${k}_{z}$ and $\eta$ (${\theta }_{i}=\theta \left(i,{k}_{z},\eta \right)$), but for simplicity, this is not included in the notation. To solve Eq. 30 for ${\theta }_{i}$, the density function $D_{a}$ (and corresponding ${uFOV}_{a}$ shape) needs to be defined.

Using Eq. 15 for the density ${D}_{a}$ of an elliptical ${uFOV}_{a}$, the incomplete elliptic integral of the first kind $F\left(\varphi , k\right)$, and ${\eta }^{\prime}=\sqrt{1-{\eta }^{2}}$, Eq. 30 can be rewritten as:

31 $$F\left( {\theta_{i} ,\eta^{\prime}} \right) = u_{i}$$

where, using the identity $F\left(\pi , k\right)\equiv 2K\left(k\right)$, ${u}_{i}$ is defined as:

32 $$u_{i} = \frac{i}{{N_{\theta va} \left( {k_{z} ,\eta } \right)}} \cdot 2K\left( {\eta^{\prime}} \right)$$

The solution for ${\theta }_{i}$ in Eq. 31 can be expressed analytically by means of the Jacobi elliptic amplitude function ${\mathrm{am}}\left(u,k\right)$:

33 $$\theta_{i} = {\mathrm{am}}\left( {u_{i} , \eta^{\prime}} \right)$$

### $\boldsymbol{\hat{u}_{i}^{*}}$*for elliptical FOV with pseudo golden angle*

For pseudo golden angle sampling [9–11], ${\hat{u}}_{i}$ in Eq. 20 is defined as:

34 $$\hat{u}_{i}^{*} = \frac{{{\mathrm{round}}\left( {N_{\theta va} \left( {k_{z} ,\eta } \right) \cdot \frac{i}{\tau }} \right){ }}}{{N_{\theta va} \left( {k_{z} ,\eta } \right)}} \cdot 2K\left( {\eta^{\prime}} \right)$$

For a circular uFOV ($$\eta =1, {\eta }^{\prime}=0$$), Eqs. 20 and 34 reduce to $$\hat{\theta }_{i}^{*} = \hat{u}_{i}^{*} = \frac{{{\mathrm{round}}\left( {N_{\theta v} \left( {k_{z} } \right) \cdot {\raise0.7ex\hbox{$i$} \!\mathord{\left/ {\vphantom {i \tau }}\right.\kern-0pt} \!\lower0.7ex\hbox{$\tau $}}} \right){ }}}{{N_{\theta v} \left( {k_{z} } \right)}} \cdot \pi$$ for VSOS, and for conventional SOS they reduce to $$\hat{\theta }_{i}^{*} = \hat{u}_{i}^{*} = \frac{{{\mathrm{round}}\left( {N_{\theta c} \cdot {\raise0.7ex\hbox{$i$} \!\mathord{\left/ {\vphantom {i \tau }}\right.\kern-0pt} \!\lower0.7ex\hbox{$\tau $}}} \right){ }}}{{N_{\theta c} }} \cdot \pi$$, which are the angles for conventional pseudo golden angle sampling.
